# Understanding Therapeutic Change Process Research Through Multilevel Modeling and Text Mining

**DOI:** 10.3389/fpsyg.2019.01186

**Published:** 2019-05-29

**Authors:** Wouter A. C. Smink, Jean-Paul Fox, Erik Tjong Kim Sang, Anneke M. Sools, Gerben J. Westerhof, Bernard P. Veldkamp

**Affiliations:** ^1^Psychology, Health & Technology, University of Twente, Enschede, Netherlands; ^2^Research Methodology, Measurement & Data Analysis, University of Twente, Enschede, Netherlands; ^3^Netherlands eScience Centre, Amsterdam, Netherlands

**Keywords:** therapeutic change processes research (TCPR), multilevel models (MLMs), text mining, process data, online interventions, text variables

## Abstract

Online interventions hold great potential for Therapeutic Change Process Research (TCPR), a field that aims to relate in-therapeutic change processes to the outcomes of interventions. Online a client is treated essentially through the language their counsellor uses, therefore the verbal interaction contains many important ingredients that bring about change. TCPR faces two challenges: how to derive meaningful change processes from texts, and secondly, how to assess these complex, varied, and multi-layered processes? We advocate the use text mining and multi-level models (MLMs): the former offers tools and methods to discovers patterns in texts; the latter can analyse these change processes as outcomes that vary at multiple levels. We (re-)used the data from Lamers et al. (2015) because it includes outcomes and the complete online intervention for clients with mild depressive symptoms. We used text mining to obtain basic text-variables from e-mails, that we analyzed through MLMs. We found that we could relate outcomes of interventions to variables containing text-information. We conclude that we can indeed bridge text mining and MLMs for TCPR as it was possible to relate text-information (obtained through text mining) to multi-leveled TCPR outcomes (using a MLM). Text mining can be helpful to obtain change processes, which is also the main challenge for TCPR. We showed how MLMs and text mining can be combined, but our proposition leaves open how to obtain the most relevant textual operationalization of TCPR concepts. That requires interdisciplinary collaboration and discussion. The future does look bright: based on our proof-of-concept study we conclude that MLMs and text mining can indeed advance TCPR.

## 1. Introduction

Traditional forms of psychotherapy are nowadays increasingly supplemented by online interactions: it is not uncommon that a counsellor seeks contact with a client through e-mail, text, chat, or other text-bearing messages. As the contact between counsellor and client becomes increasingly digitally mediated, it should be possible to trace the factors that contributed to the beneficial outcome of treatment back to these textual interactions.

In this light, the field of Therapeutic Change Process Research (TCPR) re-establishes its importance. TCPR aims to identify the mechanisms through which psychological treatments bring about positive and therapeutic change (Greenberg, 1986; Orlinsky et al., 2004; Elliott, 2010, 2012). TCPR has a long-standing tradition of studying the linguistic “products” of therapy (e.g., homework exercises, diaries, transcripts) in order to understand therapeutic change (Kazdin and Nock, 2003; Imel et al., 2015).

The rising popularity of Internet-based interventions (cf. Hoogendoorn et al., 2017) allow researchers to ask new TCPR research questions and re-establish the relevance of several known questions. Questions pertaining to the change processes that are beneficial to clients necessitate investigation of the “active ingredients” of therapy, of which many are linguistic (Muntigl and Horvath, 2005; Imel et al., 2015). TCPR has thus the potential to reveal the fundamental processes that are related to change. Aside from insight in what helps patients improve their functioning and reduce (clinical) symptoms, the importance of TCPR is also related to the rising number of people diagnosed with mental health disorders (see e.g., Andrade et al., 2013; Whiteford et al., 2013).

Over many decades, researchers attempted to answer TCPR questions; Orlinsky et al. (2004) estimated that there are more than 2000 published process-outcome studies of psychotherapy. Crits-Christoph et al. (2013) discuss several (methodological) issues related to TCPR, and express that “individual psychotherapy is not based just on an individual: it is a dyadic relationship consisting of a patient and therapist.” Similar to Kenny and Hoyt (2009) and Crits-Christoph et al. (2013) argued that—from a statistical point of view—patients are nested within their therapist, hence, TCPR is concerned with *multi-level models* (MLMs; also known as hierarchical linear models, mixed models, random coefficient, or random effects models).

Yet, we found few studies that applied MLMs specifically to study therapeutic language. In the current work, we will present an approach for the study of therapeutic change processes based on text mining and MLMs by (re-)analyzing e-mails send between counsellor and client (Lamers et al., 2015). We do so by first making a comprehensive argument for the importance of understanding multi-layered change processes (Knobloch-Fedders et al., 2015), and argue for the use of text mining to study TCPR.

### 1.1. TCPR: Therapeutic Change Process Research

Progress in psychotherapy research is not made by only demonstrating the (average) effectiveness of a treatment; the history of psychotherapy research is marked by a gradual increase in the understanding of psychotherapeutic change processes (Orlinsky et al., 2004; Braakmann, 2015). Hence, psychotherapy benefits from a greater understanding of TCPR^1^, which is defined as the scientific investigation of what occurs during psychotherapy, with regard to its clinical meaningfulness; in other words, it investigates the process through which clinically relevant changes occur within psychotherapy (Gelo and Manzo, 2015, p. 248).

Questions concerning the underlying processes that benefit the client also align with the interests of many clinical practitioners (Norcross and Wampold, 2011): *what* treatment, by *whom*, is most effective for *this* individual with *that* specific problem, and under *which* set of *circumstances* (Paul, 1967, p. 111)? Studies aimed at demonstrating average effects at group level fail to show what aspects of the intervention are related to the change the intervention realized (Barkham et al., 1993; Nock, 2007). Still, more effort is devoted to the analysis of the outcomes of psychotherapeutic interventions.

#### 1.1.1. TCPR and the Study of the Therapeutic Conversation

As early as Freud's talking cure, the importance of looking at language to understand the therapeutic process has been recognized. Conversation is still the interactive medium central to most forms of psychotherapy (Muntigl and Horvath, 2005). The idea that the verbal exchange between counsellor and client contains important ingredients of therapy fueled TCPR (Greenberg, 1986; Hill and Lambert, 2004; Elliott, 2010), which is known for its a long-standing tradition of studying the linguistic “products” of therapy (e.g., homework exercises, diaries, transcripts) in order to understand therapeutic change (Gelo et al., 2015, p. 303, 392). For example, the *Narrative Processes Coding System* is “focused on the strategies and processes by which a client and counsellor transform the events of everyday life into a meaningful story that both organizes and represents the client's sense of self and others in the world” (Angus et al., 1999).

Another reason to specifically choose text over other types of TCPR data is that a valid understanding of psychotherapeutic processes require measurements collected from multiple perspectives, including that of the client, counsellor, and (possibly) external observers (Knobloch-Fedders et al., 2015). A good way to do so is to study the text-based representation of the therapeutic interaction. Because these transcripts are a direct observation of the therapeutic process, they reflect what actually happened in therapy. Transcripts can thus provide the basis to obtain the perspective of the client, counsellor, and (or independent) observer on the therapeutic process. Such interpretations are usually measured by questionnaires or interviews, and are retrospective reflections. Transcripts come with the additional benefit that they are relatively straightforward to obtain after providing transcripts of the therapeutic conversation.

In this light, it is not unsurprising to see TCPR moving toward online interventions. Aside from being cost-efficient, web-based self-help interventions directly produce the textual interaction between therapist and counsellor, and come with the additional benefits that they are effective (see e.g., Andrews et al., 2004, 2010; Andersson et al., 2014), and easily accessible by large groups of people (Wang et al., 2007; Hoogendoorn et al., 2017). Just like transcripts, assessment of the interaction between counsellor and client in a web-based intervention has the potential of being a direct observation of the therapy process (Pennebaker et al., 2003; Schegloff, 2007; Elliott, 2012; Gelo et al., 2012).

Transcription and manual analyses mark the labor-intensive nature of TCPR, which is also the main reason why the field did not yet reach its full potential (Smink et al., under review). Traditional research methods start with the recording and transcription of a psychotherapeutic intervention so that human raters can (manually) code and analyse these transcripts (Atkins et al., 2014). Because the understanding of change processes mainly relies on qualitative analysis, these methods are only as fast as the researcher(s) conducting the research, which in practice limits their use to small scale studies (Atkins et al., 2012; Imel et al., 2015).

To strike a balance between TCPR's ambition to unravel the black-box through which therapy attains its effects and the labor-intensity of the TCPR methods, we propose to use automated text analysis methods. Text mining, a computational approach to text analysis, can be used to automatically extracted text features that can contribute to the understanding of the active ingredients of therapy. We are observant of the criticisms that algorithms have yet to achieve the same depth of analysis as humans. However, in our view, it would be a shortcoming to TCPR's ambitions if the insights that basic text features can offer remain unused. In the next session we will discuss how text mining can scale up TCPR by finding text-based predictors—also known as input variables or independent variables—from therapy related texts. We will do so making use of *multi-level models* (MLMs), an advanced statistical model that is able to capitalize on the hierarchical structure of text data.

### 1.2. Text Mining: Scaling Up TCPR

As language is an important mediator of psychotherapeutic processes, obtaining information about these processes through texts is one of the first applications of text mining. Mergenthaler (1996) compared five computer-assisted measures for the analysis of textual data of two psychotherapies, and was among the first to apply text mining for psychology. He used text mining, which he then called “computer assisted analysis of textual data,” to identify turning points in sessions, which could then be explored more deeply by humans through (qualitative) analyses methods. Anderson et al. (1999) developed *Computer Assisted Language Analysis System* (CALAS) to examine the relationship of various linguistic measures to outcome measures in high and low verbalized affect segments. Many applications of text mining are still centered around finding key moments in the therapeutic process (cf. Lepper and Mergenthaler, 2005; Pfäfflin et al., 2005; Fontao and Mergenthaler, 2008), which is also a common approach in TCPR (e.g., the “Significant Events Approach” in Elliott, 2010).

Practically, typical text mining approaches in psychology include counting words, identifying topics, and coupling the terms to a domain-specific ontology (Hoogendoorn et al., 2017). Text mining^2^ refers to a general methodological framework that includes several automated methods to analyse large corpora of texts (cf. Jurafsky and Martin, 2017). As text mining is a methodological framework that combines and includes numerous techniques and methods from many disciplines, it is not surprising that terms referring to the automatic extraction of information from text are used sometimes interchangeably, such as text mining and NLP.

#### 1.2.1. Text Mining Emotions

The Linguistic Inquiry and Word Count (LIWC) software by Pennebaker et al. (2015a) is used by many researchers, and has showed to be effective in predicting therapist empathy (Gibson et al., 2015), counsellor behavior (Pérez-Rosas et al., 2017), and identifying emotional and cognitive process in psychotherapy (McCarthy et al., 2017). LIWC categorizes word usage by counting the percentage of words that reflect—among other categories—thinking styles, emotional states, and social concerns (Pennebaker et al., 1997; Hirsh and Peterson, 2009; Tausczik and Pennebaker, 2010). LIWC taps into the underlying idea that word use is one of the most direct means of expressing thoughts and feelings (Fast and Funder, 2008), as the way individuals talk and write provides a window into their emotional and cognitive worlds psychological characteristics (Pennebaker et al., 2003, 2015b).

The writing intervention by Lamers et al. (2015) focused on different life themes, with one theme central to each of the seven modules. By asking clients to describe specific positive and several difficult memories, clients adjusted their life stories step-by-step by integration of these memories. Lamers et al. (2015) did not study the content of the e-mails.

Previous studies showed that positive therapeutic outcomes from writing interventions are associated relatively high rate of positive emotion words, few negative emotion words, and with an increasing number of “cognitive”^3^ words throughout the intervention (e.g., Campbell and Pennebaker, 2003; Pennebaker et al., 1997, 2015b). As the intervention of Lamers et al. (2015) focuses specifically on positive and difficult memories and emotions with the aim of integrating these two, we study words the reflective of these aspects in e-mails. As the intervention by Lamers et al. (2015) aims to improve integration of positive and negative memories, we expect that LIWC's “cause” and “insight” categories are mostly reflective of that process. We aim to find further evidence for these findings in data from Lamers et al. (2015), by relying on text mining and multi-level models.

#### 1.2.2. Text Mining and MLMs

Although the idea to relate words or textual aspects (in psychotherapeutic texts) to outcomes is well-established in TCPR, there are methodological issues that are specifically relevant when analyzing text data. Studying change processes in e-mails mandates accounting for the *dyadic* relation (Crits-Christoph et al., 2013, p. 301), and is therefore dependent on both the counsellor and client.

While the assumption of *independence of observations* is the basis for traditional statistical models, such as the ANOVA or regression model, some text mining models relax this assumption. For example, the naive Bayes classifier *assumes* independence assumptions between observations. The model classifies units to the category that has the highest probability; a common application of the model is the spam-filter, where e-mails are classified as either spam or “ham” (no-spam). He et al. (2012) used naive Bayes to find words that could discriminate between texts written by soldiers with or without PTSD.

Naive Bayes is a family of algorithms based on the assumption that the value of a particular (text)feature is independent of the value of any other feature. This independence assumption is too strong (“naive”); in reality, independence does not hold for texts that are written by the same person. In doing so, the model “naively' neglects the nesting of e-mails within person, ignoring the assumption of independence. In the next section work, we will argue for the importance of applying MLMs to analyse textual data for correct statistical inference, as MLMs do not violate the non-independence in e-mail data (Kenny et al., 2002). A consequence of failing to recognize the nested and hierarchical structures in e-mails is that standard errors of the estimated coefficients are underestimated, leading to an overstatement of statistical significance. MLMs recognize the existence of hierarchies in data by allowing for residual components at each level of the hierarchy.

#### 1.2.3. Psychotherapy As a Multi-Leveled Procedure

Because MLMs offer the possibility to include predictors at the level of the individual, the group and at any other level of organization, the model arises quite naturally for TCPR (Raudenbush and Bryk, 2002). Many individual change phenomena can be represented through a two-level hierarchical model. The first level represents each clients' development by an individual growth trajectory that depends on the repeated measures for each client. The second level unit represents variables that are not repeatedly measured, such as gender, income, or depressive symptoms. The first level consists out of—for example—experienced pain at the beginning, middle, and at the end of therapy. The second level consists of the clients themselves, who could be (at a third level) nested within their therapist, for examples see Baldwin et al. (2007) and (Baldwin and Imel, 2013).

From a statistical viewpoint, TCPR practically equates to research questions concerning either a (longitudinal) development over time (Crowder and Hand, 1990; Baldwin et al., 2007; Nissen-Lie et al., 2010; Fitzmaurice et al., 2011; Adler, 2012), an (dyadic) interaction between a counsellor and its client (Tasca and Gallop, 2009; Kenny and Hoyt, 2009; Crits-Christoph et al., 2013), or to both. MLMs are—compared to traditional statistical methods—particularly useful to both of these situations as they capitalize on hierarchically organized data. Many kinds of data, including observational data collected in the human and biological sciences, have a hierarchical or clustered structure.

Considering that the psychotherapeutic practice is a multi-leveled procedure, it becomes apparent that client and counsellor are the two pre-eminent levels of organization. As counselors (almost) always treat more clients, clients could be viewed as grouped within their counsellor, similar to the students being *nested* within their class (Kenny and Hoyt, 2009; Crits-Christoph et al., 2013). Crucial to any MLM is that the unit of analysis at the lowest level (the students or clients) are nested within higher level units (classes or counsellor), that itself could also be nested within (higher) even higher units (schools, therapeutic practices, or clinical institutions).

Many of the applications of MLMs in psychotherapy resolve around the question of how to assess psychotherapeutic effectiveness. Adelson and Owen (2012) examined the influence of psychotherapists on clients' clinical outcomes. Baldwin et al. (2007) and Marcus et al. (2009) both showed that higher rates of therapeutic alliance could be relate to better therapeutic outcomes through MLMs (Crits-Christoph et al., 2013). Baldwin and Imel (2013) searched the literature for studies comparing outcomes of therapists. Nissen-Lie et al. (2010) accounted for variation in early patient-rated alliance by means of various self-reports of therapists providing treatment in a naturalistic outpatient setting.

### 1.3. Research Questions

Online a client is treated essentially through the language their counsellor uses, therefore the verbal interaction contains many important ingredients that bring about change. TCPR faces two challenges, first, how to derive meaningful change processes from (the) large bodies of texts (that online interventions produce)? Second, how to assess these complex, varied, and multi-layered processes? These two questions are intimately linked: insight in complex change processes gives an indication of how to derive other meaningful processes, and visa-versa.

We therefore advocate the combination of text mining and MLMs: the former offers tools and methods to discover patterns and trends in texts; the latter can analyse processes that vary at multiple levels. As the study by Lamers et al. (2015) is a writing intervention of which the writing assignment, the e-mails themselves, and the outcomes of the intervention are available, we give a proof-of-concept based on data from this study.

## 2. Methods

### 2.1. Participants

The dataset derived from 174 clients who were recruited by Lamers et al. (2015) through advertisements in Dutch newspapers and websites. Only participants who felt depressed and were interested in writing about their life were included by Lamers et al. (2015). The sample was thus a self-selected group of individuals who had expressed interest in the program.

All participants had moderate depressive symptomatology and were randomly allocated to either the life-review “the stories we live by” (auto-biographic writing; AW), or the “expressive writing” (EW) intervention, or a waiting list condition. The mean age of the participants in the AW condition was 57.7 (*SD* = 10.3) years old, and the majority was female (75.9%). The mean age in the EW condition was 56.8 (*SD* = 7.9), and the majority was female (77.6%). In both conditions, the majority of the participants received a higher form of education (i.e., universities or colleges; AW: 48.3%, EW: 37.9%). For more details see Lamers et al. (2015).

### 2.2. Design

#### 2.2.1. Study by Lamers et al. (2015)

##### 2.2.1.1. Auto-biographic writing (AW)

The AW condition was a life-review self-help intervention that consisted of homework assignments, divided over modules that had to be completed over the course of 10 weeks. Clients communicated about their progress with trained counselors through a weekly e-mail interaction. According to Lamers et al. (2015) the self-help model program was based on insights from the autobiographical memory (Serrano et al., 2004; Brewin, 2006; Williams et al., 2007), narrative therapy (White and Epston, 1990; White, 2007), and life-review (Butler, 1963; Birren and Deutchman, 1991; Haight and Webster, 1995; Bluck and Levine, 1998; Westerhof et al., 2010b), and has been shown effective in previous studies (Korte, 2012; Westerhof et al., 2017).

##### 2.2.1.2. Expressive writing (EW)

According to Lamers et al. (2015) the EW intervention was based on the method of expressive writing (Pennebaker et al., 1997). The method consisted of daily writing about emotional experiences, for 15−30 min on 3−4 consecutive days during 1 week. Lamers et al. (2015) extended and adapted this method to an intervention with seven modules, to make it a comparable with the life-review intervention.

#### 2.2.2. Current Study

Our first intention was to demonstrate how text mining can be used to obtain change processes from e-mails. Lamers et al. (2015) concentrated their efforts on the analysis of the outcomes of the interventions but did not analyse the content of textual characteristics of the e-mails. After pre-processing, we obtained the insight, cause, positive, and negative emotion words from the LIWC program.

Our second intention was to demonstrate how multi-level models (MLMs) can be used to assess text-based measures of e-mails to aid understanding of the change processes. Similar to Lamers et al. (2015) we used the post-treatment measurement of the CES-D scale as the main outcome variable.

### 2.3. Materials

#### 2.3.1. Questionnaires

The data available to us included the pre- and post-therapeutic measurements of the CES-D. The Center for Epidemiologic Studies Depression Scale (CES-D) is a brief self-report questionnaire to measure severity of depressive symptoms in the general population (Radloff, 1977). Lamers et al. (2015) used the Dutch version of the CES-D (Beekman et al., 1997); higher CES-D scores indicated more depressive symptoms (20 items, range 0−60, α = 0.78).

The intervention of Bohlmeijer and Westerhof (2010) teaches participants about autobiographical reasoning by specifically improving the ability to reason about the autobiographical self (Lamers et al., 2015). This form of reasoning describes the process of relating episodic memories to the conceptual self (Pasupathi and Carstensen, 2003; Thorne et al., 2004). By making the moral of an individual's life-story explicit, (s)he obtains insight in what the particular memory could reveal, explain, cause, give insight, or provide a (life) lesson learned about the (autobiographical) self. These processes are extensively researched by—for example—Pennebaker and Chung (2011), mainly in the context of showing how analog experiences, such as emotions, are translate to digital forms that bear meaning, such as of stories.

This process is operationalized by phrases that LIWC analyses can detect from the *insight* (e.g., “I now realize that…”) and *cause* (e.g., “I understand why…”; Pennebaker and Chung, 2011). As the increase in insight and cause words are intractly related to emotional writing, we also study the (increase in) positive words, and (decrease in) negative words from LIWC (Westerhof et al., 2010a; Pennebaker and Chung, 2011).

#### 2.3.2. Software

We used the LIWC software of Pennebaker et al. (2015b) to analyse the e-mails for the emotion and insight categories. We used the NLTK library of Bird et al. (2009) in the programming language Python (Python Software Foundation, 2018, version 3.6), for pre-processing and dividing the e-mail texts in words and sentences.

For our statistical analyses, we relied on the programming language R (R Core Team, 2019, version 3.5.1). We used package lme4 for estimation and evaluation of our MLMs (Bates et al., 2015), and package psych for making descriptions of our variables (Revelle, 2018).

### 2.4. Data

#### 2.4.1. Available Data

The data included the pre- and post-therapeutic measurements of the CES-D scale, and the e-mails exchanged between counselors and clients (2079 e-mails in total).

#### 2.4.2. Complete Cases

In total, data of 174 clients was available to us from Lamers et al. (2015). We only used clients with no missing data. 166 of the 174 clients (95.4%) had a complete CES-D score. Not all e-mails were available, so we could only analyse the e-mails of 104 clients (59.8%). After removing duplicates, we included 97 clients in our analyses (55.7%, all percentages calculated against the original total of 174 clients).

#### 2.4.3. Anonymization

Identifying information has been removed from the dataset that contained the outcomes (“structured” data), we identified clients based on a unique four digits number. The e-mails (“unstructured” data) have been anonymized by removing all (e-mail) addresses, phone numbers, names of persons, organizations, and locations. Client names and counsellor names have been replaced by the previously mentioned unique four digits number so that it remained possible to identify which mails were written by the same person and which clients were treated by the same counsellor. The counselors were also anonymized.

#### 2.4.4. Process Data

The e-mails of Lamers et al. (2015) should include the whole therapeutic process because they are the only form of interaction between counsellor and client. The e-mail procedure is explained (in Dutch) in detail in Bohlmeijer and Westerhof (2010). We will give some quotes that we translated from Dutch to English to give an impression of process data in a therapeutic context.

The first quote comes from a female participant: “*My trust in people is damaged pretty badly, I'm no longer in such good faith as I was in the past*.” In response, the counsellor asks: “*Can you tell us a bit more about this? How did this happen? Are there times when you feel that you can trust people?*”

In the second week a male participant writes: “*By writing about myself, and especially naming the nice aspects about my life, I notice that writing is already paying off*.” In the sixth week he writes: “*I feel that I am coming back to who I am*.” He also expresses his graduate toward the counsellor: “*I do not have a specific question for you, a reaction from you based on my writing already is already enough. However, if you do ask questions, that would help me even further*.”

The third example comes from a (different) female participant: “*How should I continue with my life? Is it okay? Almost thirty years ago I lost my brother and my sister-in-law. I lost my 10-year-old daughter…Losing a child is pretty much the worst thing that can happen to you*.” In week seven she wrote (about her daughter): “*The tears are rolling down my cheeks as I think about you intensively. Over the duration of the course I have learned to balance between positive and negative emotions by means of communication or through writing. I succeeded, because I know that you knew that I am still an optimist in life. You and dad have a share in this. You were both never judgemental, but always stimulating*.”

### 2.5. Procedure

#### 2.5.1. Selection of the Text Variables From LIWC

We chose to use the number of *insight* and *cause* words from the cognitive process category, and the number of *positive* and *negative* words form the LIWC program (Pennebaker et al., 2015b). We had several reasons for doing so, first of all, past studies showed that positive therapeutic outcomes are associated with writing assignments of individuals that include relatively high rates of positive emotion words, few negative emotion words, and with an increasing number of cognitive words throughout the intervention (Pennebaker et al., 1997, 2015b; Campbell and Pennebaker, 2003; Campbell et al., 2013). Secondly, these basic text features are—as the name implies—relatively straightforward to obtain from an e-mail. Third, it is our ambition to show how textual information can be *obtained* through text mining and *analyzed* with MLMs. We do not aim to advance TCPR theory in our current paper: determining which textual predictors are meaningful is beyond the scope of our work. We intend to show how TCPR can be modeled in e-mails. Lastly, by bridging text mining and MLMs other TCPR researchers are enabled to advance TCPR theory using these two methodologies.

#### 2.5.2. Pre-processing

We used the NLTK library to preprocess the e-mails. NLTK pounts sentences by counting word-terminal end-of-sentence punctuation like the period, question mark and / or exclamation mark. NLTK has a limited list of abbreviations, which are not included in the punctuation/sentence count. Word-internal punctuation, like the first period in e.g., is ignored. Handling of interjections depends on their punctuation, for example, “Oh?” is a separate sentence while “Oh,” is part of the following sentence. Sentence fragments and quotes with end-of-sentence punctuation are counted as separate sentences.

NLTK is an often used Python library for text pre-processing, as it provides detailed documentation in Bird et al. (2009) on the order and content of the preprocessing steps.

#### 2.5.3. Pre- and Post-therapeutic Measurements of the Text-Variables

We calculated the pre- and post-therapeutic scores of the text-variables (*insight, cause, positive*, and *negative* words form LIWC program) by averaging over the number of these words as counted by Pennebaker et al. (2015b) in the first and last three e-mails of the intervention by (Lamers et al., 2015). The original intervention also included a third time-point (*T*_0_ a depression measure at the onset of the writing treatment, *T*_1_ a measure at the end of the treatment, and *T*_2_ a follow-up measure). However, only for the first two measurements (those at the beginning and end of therapy) we had e-mail data available. Hence, we dropped the follow-up measure (*T*_2_) from our dataset, as we could not use in our text mining models.

### 2.6. Analyses

**Figure 1:**
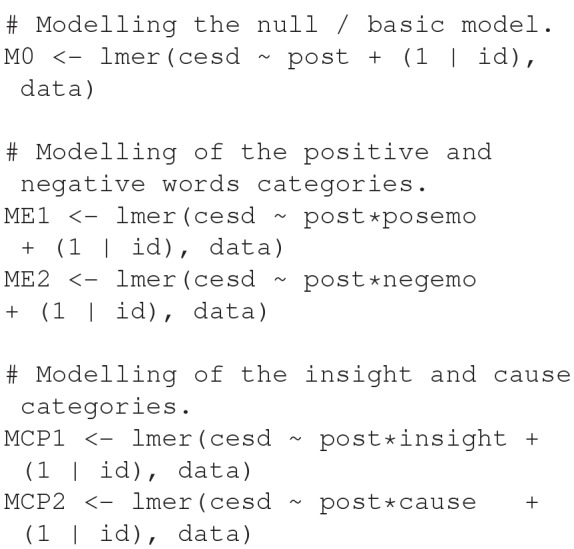
R code of the five multi-level models (M0, ME1, ME2, MCP1, and MCP2) using package lme4. In all models, we estimated the post-therapeutic measurement of CES-D (cesd) based on a random intercept for each client (id). In ME1 we estimated the post-therapeutic effect of the number of positive emotion words as the interaction effect between the number of positive emotion words (posemo) and an indicator variable (post). The other models are similar, in ME2 we estimated the effect of the number of negative emotion words (negemo), in MCP1 we estimated the effect of the number of insight words, and in the MCP2 we estimated the effect of the cause words. M0 is nested under each of these models.

In total, we estimated five MLMs, see Figure 1 for an overview and the R code. The regression equations below will give an indication of how the R code and equations are related. The data we used were the pre- and post-therapeutic measurements of the CES-D and the *insight, cause*, and the *positive* and *negative emotion* words of the LIWC Pennebaker et al. (2015b).

The pre- and post-therapeutic measurements of the CES-D scale were considered to be an outcome variable of the MLMs. Each MLM had a random intercept for the client to describe the variability in outcome scores across clients. An index *i* is used to refer to a pre-therapeutic score (*i* = 1) or post-therapeutic score (*i* = 2), and an index *j* is used to refer to the j^th^ client. Then, the outcomes can be described with a MLM, which is represented by

(1) $$\begin{array}{l} {\text{CES-D}}_{ij}=\mu +u_{0j}+{\text{X}}_{ij}\beta_{1}+e_{ij}. \end{array}$$

The errors *e*_*ij*_ are assumed to be normally distributed with a mean of zero and variance $\sigma_{e}^{2}$, and the random intercepts *u*_0*j*_ is also assumed to be normally distributed with mean zero and variance τ. The parameter μ is the general mean across scores. The predictor variables are stored in a matrix **X**. The common effects, β_1_, represent the effects of the predictor variables on the outcomes CES-D. The predictor variables **X** explain variance in scores across the pre- and post-therapeutic measurement, and do not explain any change between the pre- and the post-therapeutic scores. To assess change, an indicator variable is used for the post-therapeutic measurement with *D*_1*j*_ = 0 for all the pre-measurements, and *D*_2*j*_ = 1 for the post-measurements. A significant interaction between the post-therapeutic measurement scores and a predictor variable would identify a change.

The MLM described in Equation (1) can be recognized as a repeated measures model, where the model describes the profile of two measurements for each subject. The well-known models for pre- post-therapeutic measurements are the change-score model (the difference in outcomes is regressed on the predictor variables) and the regressor variable method (ANCOVA; the post-therapeutic measurement is regressed on the pre-therapeutic measurement and predictor variables, e.g., Allison, 1990). Allison (1990) and Kutner and Brogan (1982) showed that the repeated measures model is more general than the change score model, which is more restrictive and provides less information about the data. Furthermore, it is possible to control for additional group differences at the pre-therapeutic measurement by including additional predictor variables (Schmidt et al., 2016). This can be beneficial for instances when different groups have not been randomly assigned to different treatments and pre-therapeutic measurement differences between groups need to be accounted for to measure treatment effects. According to the repeated measures model, the MLM for the CES-D scores using the post-therapeutic measurement indicator *D* is given by,

$$\begin{array}{l} {\text{CES-D}}_{1j}=\mu_{0}+u_{0j}+{\text{X}}_{j}\beta_{1}+e_{1j}\\ {\text{CES-D}}_{2j}=\mu_{0}+u_{0j}+{\text{X}}_{j}\beta_{1}+\mu_{1}I\left(D_{2j}=1\right)\\ \;+{\text{X}}_{j}I\left(D_{2j}=1\right)\beta_{2}+e_{2j}. \end{array}$$

The parameters β_1_ represent the common effects of the predictor variables **X** on the outcomes CES-D and explain part of the common variance in the pre- and post-therapeutic measurements. The intercept μ_0_ represents the average score level at the pre-therapeutic measurement, and the μ_1_ the average change in scores between the pre- and post-therapeutic measurements. Given the effects of the predictor variables, the μ_1_ represents the assessed average change in measurements that is not explained by any predictor variable. The parameters β_2_ represents the contribution of the predictor variables in explaining unique variance in the post-therapeutic measurement scores. Significant interaction β_2_ effects identify and explain a change in scoring between the pre- and post-therapeutic measurements.

The first model, our “null” model, acted as a baseline, hence the name M0. In M0, we test whether a random intercept for each client explains variability in outcome scores across clients. In ME1, we test whether the text-predictor variable positive emotion words contributes to explaining the unique variance in post-therapeutic scores. In ME2, MCP1, and MCP1 we test similar hypotheses, but then with the number of the number of negative emotion, insight and cause words.

## 3. Results

We intended this section as a guideline for TCPR researchers who aspire to use text mining for multilevel modeling. We start with a statistical summarization of the variables that we used in our five multilevel models. Then we present and interpret the fixed and random effects of these models, and the corresponding goodness of fit measures. In doing so, we hope to give guidance of how these two frameworks should be combined, without presenting results of statistical significance.

### 3.1. Variable Descriptions

In total, we used five variables, one from the intervention from Lamers et al. (2015); we obtained the other four (text) variables from the LIWC program by Pennebaker et al. (2015b). The CES-D score (*M* = 19.42, *SD* = 8.75), the number of positive emotion words (*M* = 40.25, *SD* = 27.15), the number of negative emotion words (*M* = 21.62, *SD* = 11.73), the number of insight words (*M* = 48.19, *SD* = 28.49), and the number of cause words (*M* = 20.93, *SD* = 14.1) are summarized in Table 1 (mean and standard deviations in the text are combinations of the pre- and post-therapeutic measurements).

**Table 1:** Descriptive statistics of the CES-D score, insight, cause, positive, and negative emotion words from the e-mails of the clients on the pre- (*T*_0_) and post-therapeutic (*T*_1_) measurement.

**Variable**	**Time**	***M***	***SD***	**Median**	**Min**.	**Max**.
CES-D	*T*_0_	23.41	7.51	23	10	49
	*T*_1_	15.42	8.07	14	1	37
Positive emotion	*T*_0_	36.78	20.73	35	2	110
	*T*_1_	43.71	32.07	34	0	162
Negative emotion	*T*_0_	25.47	16.17	22	1	77
	*T*_1_	17.76	13.76	14	0	62
Insight	*T*_0_	50.52	27.03	50	1	142
	*T*_1_	45.86	29.84	41	2	173
Cause	*T*_0_	21.32	12.92	18	2	59
	*T*_1_	20.54	15.25	18	0	85

### 3.2. Multilevel Models

In total, we estimated five multilevel models (see Figure 1). The post-therapeutic measurement of CES-D was the main outcome. In M0, model 0, we estimated the post-therapeutic measurement based on a random intercept for each client. M0 is nested under the other four models. In ME1 (“Model *Emotion*”), we estimated the post-therapeutic effect of the number of positive emotions words and a random intercept for each client. ME2 was similar to ME1, but instead of positive emotion words, we estimated the effect of (the number of) negative emotions words. MCP1 (“Model *Cognitive Process*”) was similar in the same respect: we estimated the effect of insight words (instead of positive or negative words), and in MCP2 we estimated the effect of cause words.

### 3.3. Interpretation

The data do not support our hypotheses that the writing intervention improves the number of positive, insight and cause words, while decreasing the number of negative words. Rather than using the data of Lamers et al. (2015) as a case to obtain new insights about TCPR, we present it as a use case for process researchers who wish to investigate e-mail data through multilevel models. Accordingly, we assessed the results in Table 2 in four steps.

**Table 2:** Model fit, parameter estimates and corresponding standard errors of the fixed and random effects of the five multilevel models.

	**Baseline**		**Emotion**				**Cognitive processes**			
	**M0**		**ME1**		**ME2**		**MCP1**		**MCP2**	
*Fixed*	23.41 (0.792)	^*^	22.83 (1.516)	^**^	22.61 (1.397)	^**^	21.62 (1.588)	^**^	21.69 (1.447)	^**^
Intercept	-7.99 (0.858)	^*^	0.02 (0.035)		0.03 (0.045)		0.04 (0.027)		0.08 (0.057)	
Post-indicator			-5.60 (1.735)	^*^	-6.33 (1.649)	^**^	-5.33 (1.820)	^*^	-5.50 (1.663)	^**^
Variable interaction			-0.06 (0.039)		-0.08 (0.066)		-0.05 (0.033)		-0.12 (0.068)	
*Random*									
$\sigma_{e}^{2}$	35.74		35.37		35.80		35.46		35.66	
τ	25.06		24.89		25.21		25.21		24.72	
*Model fit*									
deviance	1327.38		1323.48		1325.89		1324.58		1324.24	
AIC	1335.38		1335.48		1337.89		1336.58		1336.24	
BIC	1348.45		1355.09		1357.50		1356.19		1355.85	
LogLik	-663.69		-661.74		-662.95		-662.29		-662.12	
χ^2^			3.89		1.48		2.80		3.13	
χ^2^ df			2		2		2		2	
*Effect size*									
$\Omega_{0}^{2}$ df	0.67		0.68		0.67		0.68		0.67	

*The mean of the text-variable, indicated by “variable” in the table, changes between the five models: in ME1 it is the number of positive words, in ME2 it is the number of negative words, in MCP1 it is the number of insight words, and in MCP2 it is the number of cause words. The “interaction” variable is the interaction between the text variable and the post-therapeutic indicator (“post. indi.”)*.

*Coefficients (and standard errors)*.

* *p < 0.01*.

** *p < 0.001*.

#### 3.3.1. 1. Fixed Effects: Intercept and Post-therapeutic Indicator

The post-therapeutic effect of the writing intervention is estimated as the interaction (“interaction” in Table 2) between the model specific variable (“variable,” with a varying meaning between the models, variable indicates the number of positive emotion words in ME1, negative emotion words in ME2, insight words in MCP1, and cause words in MCP2) and the post-therapeutic indicator (“post. indi.”) in Table 2. As we are specifically interested in the post-therapeutic interaction effect, we do not interpret the effect of the model specific variable and post-therapeutic indicator in Table 2. The fixed effect of M0 is the grand mean (μ), which is interpretable as the positive effect of the writing treatment, without specific change effects of the word categories we included. We also estimated the effect of the post-therapeutic indicator. However, this effect should not be interpreted, as it merely acts as a dummy variable in our model.

#### 3.3.2. 2. Assess Post-treatment Effects

There are two ways to evaluate the model(s). The first is based on values of the post-therapeutic interactions. Table 2 does not give an indication that models ME1, ME2, MCP1, and MCP2 have significant post-treatment effects at the *p* < 0.05 level. Because all the relevant information lies in the interaction effect, the effect of the (text-)“variable” should also not be interpreted.

The second way to evaluate models is based on model fit. Of the all the model fit information in Table 2, the χ^2^-test is perhaps the most straightforward to interpret, as it comes with a significance test. As none of the χ^2^-tests are significant, the model fit information in Table 2 does not indicate that one of the four models (ME1, ME2, MCP1, and MCP2) is a (significant) improvement over the baseline model M0. The other fit criteria should be seen as measures that indicate good model fit if they are closer to zero (there are several good sources, we suggest Burnham and Anderson, 2004, as a starting point).

#### 3.3.3. 3. Random Effects

The variance of the random effect τ express the variation in post-therapeutic depression scores for individuals. The variance of the residual error $\sigma_{e}^{2}$ expresses the variance of the measurement errors, conditional on the individuals (the random effects). Table 2 shows that the main effect of the text variables are—relative to the interaction effects—quite large. This is an indication that the sample (and population) are quite heterogeneous, making it difficult to estimate the effect of the writing intervention, as homogeneous treatment effect are simpler to estimate.

#### 3.3.4. 4. Effect Size

For the calculation of the effect sizes, we followed the suggestions of Xu (2003). $\Omega_{0}^{2}$ in Table 2 is a generalization of the well-known *R*^2^ measure, which can be interpreted as a measure for explained variance in multilevel models. Overall, Table 2 shows that all models have a relative large proportion of explained variance. However, as model fit is (decimally) similar for all models, we cannot conclude that one model should be preferred over the others.

## 4. Discussion

Key questions of Therapeutic Change Process Research (TCPR) usually adhere to obtaining a thorough understanding of the change processes that are (most) beneficial to the client. For TPCR, the pertinent question is not whether psychotherapy is effective, but how change occurs. It is common for TCPR to study the language used in the (therapeutic) interaction between client and counsellor in order to obtain answers to this question. Two challenges arise, how to obtain text-measures that relate to change processes, and how to analyse these change processes. We argued that text mining could be used for the first challenge, and multi-leveled models (MLMs) to overcome the second.

### 4.1. Conclusion

The complete-data subset from Lamers et al. (2015) does not suggest that the writing intervention contributes to change in the (number of) insight, cause, positive, and negative emotion words. The analyses show that the intervention does decrease post-therapeutic depression, however, the data did not indicate that this decrease could be associated with one of the text variables.

We aimed to make a case for the correct analyses of e-mail data, by obtaining text variables from large bodies of text, not to obtain theoretical insights. We showed that text mining is an appropriate tool to model change processes, as it can answer questions related to change processes.

The second goal of our paper was to show how complex and multi-layered change processes should be assessed. We presented a straightforward re-parametrization of multi-level models, that allowed for assessing post-therapeutic change. The way we parametrize our MLMs allows for modeling a baseline (pre-therapeutic score) and change (post-therapeutic score) over time, while accounting for the dependency between pre- and post-therapeutic score of each client. This also corresponds to growth modeling of multilevel data, where measurements are nested within subjects (Muthén, 1997). The association of specific text variables to the outcomes of the intervention was illustrative for these two points. Based on this proof-of-concept, we conclude that obtaining and analyses of textual information through text mining and MLMs can indeed advance TCPR.

#### 4.1.1. Relevance

The main advantage of these models is that it opens up the possibility to engage more with clients in therapeutical settings. With online interventions on the rise, there is clear room to do so. The information from texts, which is directly accessible and does not require intensive transcription procedures, and can then be used to steer the therapeutic process in the desirable direction. Text mining can thus be used as a form of “direct feedback,” as MLMs allow for correct modeling of the relations between variables.

### 4.2. Open Challenges

We proposed that text mining can be used to identify the important change processes within therapy related texts, and MLMs can be used to explain the relations between processes and outcomes. Full demonstration of the capabilities of this framework requires multiple datasets, and many of the problems that we faced require the attention of more researchers. We start the discussion session by describing these (open) challenges. Then, in the next section, we cover the limitations specific to our study.

#### 4.2.1. Operationalization

Operationalization is one of the first challenges that users of text mining for TCPR face. Many of the TCPR constructs are theoretical, and need to be operationalized into linguistic features so that they are clearly distinguishable, measurable, and understandable in terms of empirical observations. Examples of these variables include emotional ventilation, dramatic relief, tension release, abreaction, or catharsis (for more examples, see Grencavage and Norcross, 1990). Operationalization is not only an important aspect for TCPR, nor is it limited to psychology, the whole social and life sciences require good operationalizations.

The linguistic products of therapy (diaries, psychotherapeutic assignments, or transcripts of the therapeutic interaction) provide rich source of research material, provided that the variables of interest are adjustable to texts. In our current work, we used a basic text features from LIWC. We justified our use of these basic text features because we aimed to give a proof-of-concept with the intend of showing how TCPR and MLM can be bridged.

However, our choice for such a basic text variable leaves one of the largest challenges open: what to (text) mine? Traditionally, the text mining community was more concerned with collecting, storing and managing large bodies of unstructured text rather than applying theoretical models from other fields. Advances in the field of computer science made technical issues less insurmountable than they were a decade ago (Mayer-Schönberger and Cukier, 2013, p. 8). As a results, text mining is no longer reserved for those with a computer science degree.

The increase in solved technical issues did not lead to insights in “what to mine.” We did not aim to advance TCPR theory with our current paper; we intended our work as a method paper, because with the current state of the literature, it is difficult to determine which textual predictors are meaningful. Also, we feel that our proposition to bridge text mining and MLMs itself allows for advancing TCPR theory. Constructs as described by Grencavage and Norcross (1990), Orlinsky et al. (2004), Elliott (2010), and Elliott (2012) require a ‘translation’, or adjustment, before text mining is applicable to these data types. Domain experts in the TCPR field are well-equipped to face this question, but this requires an interdisciplinary approach.

We showed how MLMs and text mining can be combined, but our proposition leaves open how TCPR concepts should be operationalized for text mining metrics. That would require an interdisciplinary collaboration and discussion. However, the future does look bright: based on our proof-of-concept study we conclude that MLMs and text mining can indeed advance TCPR.

The next step in that direction, would be to—aside from LIWC—incorporate other existing text mining software, such as TCM (*Therapeutic Cycles Model*; Mergenthaler, 1996), or CALAS (*Computer Assisted Language Analysis System*; Anderson et al., 1999).

#### 4.2.2. Measurement Error

Elliott (2010) argued that TCPR is plagued by measurement error. Although the term “error” is often used, in our experience, it can refer to two different concepts depending on the field of study. With the risk of over-generalization, in the machine learning community and other fields that rely heavily on predictive analytics, error often refers to the error or confusion matrix. The table of confusion reports the number of false positives and negatives, and the true positives, and negatives. These measurement represent the performance of an algorithm. Error then refers to measures of predictive error, the difference between the observed values and the values predicted by the model.

In statistics, error is related to measurement error, which represents the difference between a measured value of a quantity and its true value. Measurement error is often used to indicate whether or not measurement is reliable. Reliability expresses how repeatable measurements are when remeasured. The reliability of a measure is then a direct function of the amount of error is present in the measurement. Because no behavioral measure is perfectly reliable, some degree of measurement error will always occur. Therefore, reliability is low when there is a abundance of error, and vice versa. The underlying idea is that every observation is a combination of the hypothetical true score plus some measurement error.

Although nowadays ideas appear to be floating freely between machine learning and statistics (Wasserman, 2010, p. 8), some concepts—such as measurement error—are traditionally more associated with one branch rather than the other (see for example Donoho, 2017). Measurement error is well-established in statistics, and has potential for machine learning disciplines such as text mining. Variables are simply an operationalization of the process, behavior or item that we are trying to measure. Estimation of the measurement error reflects the uncertainty present in the estimate. Consistency of the research measures benefits when accounting for measurement error.

In fact, with respect to measurement error, MLMs are the way forward. MLMs recognize the existence of several levels, nesting and hierarchies in data. MLMs capitalize on this concept by allowing for the inclusion of residual components at each level of the hierarchy. Hence, the precision of the estimation of measurement error increases, as the residual variance is partitioned.

#### 4.2.3. Sample Size

TCPR is rooted in qualitative research methods; MLMs come from the quantitative sciences. Intensive case-studies are not uncommon for qualitative scientists, but will lead to statistical power issues for MLMs. As MLMs introduce multiple levels, the total number of units observed for each level become the sample size. The relevant sample size for power issues depends on the parameters that are being tested. Unlike the traditional regression, there is a difference between testing a regression coefficient or a variance parameters in a MLM.

The main limit is the sample size at the highest level of organization. Naturally, having multiple measures (at the first level) for one client (second level) is less informative then having these same multiple measures for multiple clients. The number of clients will therefore be one of the main issues for using MLMs for TCPR, but it will limit the wide scale application of MLMs for TCPR.

### 4.3. Limitations

We already gave an impression of some overarching open challenges that—in their current form—limit the applicability and wide-scale impact of the ideas we presented in the current work.

#### 4.3.1. Excluded Therapy

Based on the design of Lamers et al. (2015), it would also have been straightforward to model the effect of treatment. Modeling treatment as a random effect could have provided an insight in the efficacy of the treatment for each individual client. The fixed effect of treatment would have given some insight in the average efficacy of the treatment groups in comparison with each other.

We however, as Lamers et al. (2015), we could not differentiate between the two conditions of the treatment. They found both writing conditions to be helpful in comparison to the control group, but could not differentiate between the expressive writing and autobiographical writing conditions.

We justified our exclusion furthermore because we only intended to show that text mining can be used to obtain additional predictors for multilevel models. Our intend was not to offer new theoretical insights for psychological writing interventions; we intended to offer methodological rather theoretical insights.

#### 4.3.2. Complete Cases

We only included clients with complete cases and did not attempt to account for the missing data. First of all, it was difficult to determine why certain measurements where missing for an individual. Lamers et al. (2015) gave an overview of drop-out and missing data: it was challenging for us to determine *post-hoc* what the exact reason for missing data or drop out was for an individual based on general information.

Because we did not understand the underlying reason for the occurrence of missing data, we were hesitant in choosing an imputation technique. Also, because we did not intend to draw theoretical conclusions from our work, we felt that the issues with generalization and validity associated with ignoring missing data were less relevant for our proof-of-concept.

### 4.4. Future Research

MLMs come with the well-known advantage that the model can incorporate the hierarchical structure of the data. This is idea holds potential for TCPR, as change processes are often multifaceted and multi-layered. For example, an interesting analyses would be to see the effect counselors have on their clients. As a counsellor almost always treats multiple clients, it is possible to estimate the effect of a counsellor on its clients. Combining this form of nesting with other forms of nesting, such as the treatment effect itself, it would then be possible to estimate counsellor efficacy in different arms of the treatment. Accounting for clustering influences the estimation of the treatment effect as these influences are expressed as parameters in the model.

TCPR would also receive an enormous boost when change processes could be automatically detected through text mining. Some methods, such as the *Innovative Moments Coding Scale* (perhaps better known under its abbreviated name ICMS, see Gonçalves et al., 2009, 2010), already provided an avenue for doing so.

We are optimistic about TCPR's future through the happy marriage between text mining and MLMs. Especially in the social sciences, many phenomena can considered to be leveled, and the usage of text mining is already picking up. Social scientists in general often intend to learn about relations between variables in the population. In our view, in comparison with machine learning models, MLMs are of use to social scientists because they can provide theoretical insights in the relationships between, rather than building a black box model with the goal of attaining good predictive qualitative. MLMs can thus be used to explain relations between variables, whereas text mining can thus be used to obtain important therapy related variables, given that other TCPR research point in the direction of which important constructs are present in texts.

## Ethics Statement

We reused the data of Lamers et al. (2015). Westerhof is also a project member of the current team, so we reused his data, that already received medical ethical approval and - as can be read in the paper - was anonymized before shared with the current authors.

## Author Contributions

WS conducted the research and wrote the paper. J-PF contributed to the multilevel analyses. ET contributed to data handling in Python and pre-processing of the data. GW contributed the data. J-PF, ET, GW, AS, and BV helped with setting up project and gave feedback throughout.

### Conflict of Interest Statement

The authors declare that the research was conducted in the absence of any commercial or financial relationships that could be construed as a potential conflict of interest.

## Footnotes

^1^It should be noted that various terminologies are used in the literature, e.g., Change Process Research (CPR: Elliott, 2010; Greenberg, 2007), Psychotherapy Process Research (PPR: Gelo et al., 2012), and some of the early works simply refer to “change” (Braakmann, 2015; Hill and Corbett, 1993). The term “process-outcome research” is also often used, for example by Orlinsky et al. (2004), who defined it as “(primarily) the actions, experiences, and relatedness of patient and therapist in therapy session when they are physically together, and (secondarily) the actions and experiences of participants specifically referring to one another that occur outside of therapy sessions when they are not physically together” (Crits-Christoph et al., 2013, p. 311). To emphasize that we are dealing with change resulting from *therapy*, we propose to describe change processes as TCPR: *Therapeutic Change Process Research*.

^2^We recommend Manning and Schütze (1999); Feldman and Sanger (2007); Jurafsky and Martin (2017) for a detailed overview of text mining. For aspiring text mining practitioners, we recommend the NLTK library available in the programming language Python (Bird et al., 2009).

^3^Cognitive words are a word category from the LIWC program.
